# Functional connectivity in autism spectrum disorder evaluated using rs-fMRI and DKI

**DOI:** 10.1093/cercor/bhad451

**Published:** 2023-11-27

**Authors:** Yasuhito Nagai, Eiji Kirino, Shoji Tanaka, Chie Usui, Rie Inami, Reiichi Inoue, Aki Hattori, Wataru Uchida, Koji Kamagata, Shigeki Aoki

**Affiliations:** Department of Psychiatry, Juntendo University School of Medicine, 2-1-1 Hongo Bunkyo-ku Tokyo 113-8421, Japan; Department of Psychiatry, Juntendo University School of Medicine, 2-1-1 Hongo Bunkyo-ku Tokyo 113-8421, Japan; Department of Psychiatry, Juntendo University Shizuoka Hospital, 1129 Nagaoka Izunokuni-shi Shizuoka 410-2295, Japan; Juntendo Institute of Mental Health, 700-1 Fukuroyama Koshigaya-shi Saitama 343-0032, Japan; Department of Information and Communication Sciences, Sophia University, 7-1 Kioi-cho Chiyoda-ku Tokyo 102-8554, Japan; Department of Psychiatry, Juntendo University School of Medicine, 2-1-1 Hongo Bunkyo-ku Tokyo 113-8421, Japan; Department of Psychiatry, Juntendo University School of Medicine, 2-1-1 Hongo Bunkyo-ku Tokyo 113-8421, Japan; Juntendo Institute of Mental Health, 700-1 Fukuroyama Koshigaya-shi Saitama 343-0032, Japan; Department of Radiology, Juntendo University School of Medicine, 2-1-1 Hongo Bunkyo-ku Tokyo 113-8421, Japan; Department of Radiology, Juntendo University School of Medicine, 2-1-1 Hongo Bunkyo-ku Tokyo 113-8421, Japan; Department of Radiology, Juntendo University School of Medicine, 2-1-1 Hongo Bunkyo-ku Tokyo 113-8421, Japan; Department of Radiology, Juntendo University School of Medicine, 2-1-1 Hongo Bunkyo-ku Tokyo 113-8421, Japan; Faculty of Health Data Science, Juntendo University, 6-8-1 Hinode Urayasu-shi Chiba 279-0013, Japan

**Keywords:** autism spectrum disorder, cerebellum, diffusional kurtosis imaging, functional connectivity, rs-fMRI

## Abstract

We evaluated functional connectivity (FC) in patients with adult autism spectrum disorder (ASD) using resting-state functional MRI (rs-fMRI) and diffusion kurtosis imaging (DKI). We acquired rs-fMRI data from 33 individuals with ASD and 33 healthy controls (HC) and DKI data from 18 individuals with ASD and 17 HC. ASD showed attenuated FC between the right frontal pole (FP) and the bilateral temporal fusiform cortex (TFusC) and enhanced FC between the right thalamus and the bilateral inferior division of lateral occipital cortex, and between the cerebellar vermis and the right occipital fusiform gyrus (OFusG) and the right lingual gyrus, compared with HC. ASD demonstrated increased axial kurtosis (AK) and mean kurtosis (MK) in white matter (WM) tracts, including the right anterior corona radiata (ACR), forceps minor (FM), and right superior longitudinal fasciculus (SLF). In ASD, there was also a significant negative correlation between MK and FC between the cerebellar vermis and the right OFusG in the corpus callosum, FM, right SLF and right ACR. Increased DKI metrics might represent neuroinflammation, increased complexity, or disrupted WM tissue integrity that alters long-distance connectivity. Nonetheless, protective or compensating adaptations of inflammation might lead to more abundant glial cells and cytokine activation effectively alleviating the degeneration of neurons, resulting in increased complexity. FC abnormality in ASD observed in rs-fMRI may be attributed to microstructural alterations of the commissural and long-range association tracts in WM as indicated by DKI.

## Introduction

Autism spectrum disorders (ASDs) are a cluster of phenotypically and genetically heterogeneous neurodevelopmental disorders that are diagnosed by core deficits in social communication and the presence of repetitive, stereotyped behaviors, but have diverse genetic and environmental risk factors (Sydnor and Aldinger 2022). Individuals with ASD have been reported to have a multitude of complex brain abnormalities (McKenna et al. 2020), including early overgrowth (Dementieva et al. 2005), microstructural disorganization (Bailey et al. 1998), and deficits in both cytoarchitecture (Casanova et al. 2006) and functional connectivity (FC) (Di Martino et al. 2014). Moreover, ASD is highly heritable, but displays considerable heterogeneity with both common and rare genetic variation in hundreds of genes likely contributing to the clinical variability (Sandin et al. 2017; Dias and Walsh 2020). Convergent evidence across multiple studies and modalities demonstrates structural and functional impacts to the pathophysiology of the ASD brain, and these findings are accompanied by extreme diversity and interplay with the excitatory/inhibitory imbalance hypothesis of ASD (Sydnor and Aldinger 2022).

In terms of FC, ASDs are characterized by atypical FC within and between particular neural networks and regions in the brain (Belmonte et al. 2004; Kana et al. 2006; Kitzbichler et al. 2015), including decreased interhemispheric connectivity (Just et al. 2007; Di Martino et al. 2009; Anderson et al. 2011; Dinstein et al. 2011; Li et al. 2019) and reduced lateralization of typically asymmetrical processes (Sydnor and Aldinger 2022). Underconnectivity in some specific networks and circuitries, such as the default mode network (Kennedy et al. 2006; Kennedy and Courchesne 2008a; Kennedy and Courchesne 2008b; Lombardo et al. 2009; Monk et al. 2009; Assaf et al. 2010; Weng et al. 2010), the salience network (Uddin and Menon 2009), or self-representation circuitry (Lombardo et al. 2010), are also reported. In contrast, overconnectivity in the cerebellar network has been also reported (Nakamura et al. 2023). On the other hand, FC complexity in ASD has been characterized by some descriptions as local overconnectivity but long-distance underconnectivity (Courchesne and Pierce 2005; Cherkassky et al. 2006; Geschwind and Levitt 2007; Just et al. 2007; Rippon et al. 2007; Casanova and Trippe 2009; Kana et al. 2009) or strong activation in parietal cortex during suppression of distractors but low activity in integrative brain regions in prefrontal and medial temporal cortices (Belmonte 2004). That is, sensory inputs should evoke abnormally large activations for attended and unattended stimuli alike, reducing the selectivity, and incurring a high load at later stages of perceptual processing as distractors are differentiated from targets. Conversely, regions subserving integrative functions will be cut off from their normal inputs and should therefore manifest reductions in functional correlations with sensory regions (Belmonte et al. 2004). While these studies have suggested that functional brain connectivity is disrupted in ASDs, there is little consensus on a uniform explanatory model of functional brain atypicalities in ASD with age, sex, comorbidities, and various methodological choices likely affecting the directionality (i.e. hypo- vs. hyperconnectivity) of the observed atypicalities (Linke et al. 2017; Reiter et al. 2019; Olson et al. 2020; Nair et al. 2021).

Atypical FC may implicate white matter (WM), which contains bundles of axons that allow for fast and efficient neuronal communication (Pajevic et al. 2014). Some genetic factors might contribute to axon alterations in ASD (Hashem et al. 2020). Mutations in the chromodomain helicase DNA binding protein 8 gene, one of the most commonly reported mutations in autism, have been associated with reduced axon and dendritic growth in humans, resulting in pathophysiology of ASD such as reduced information processing speed (Wegiel et al. 2018; Xu et al. 2018). Pathway enrichment among ASD-implicated genes also suggests some functional commonalities, including impairments in synapse function and chromatin modification (De Rubeis et al. 2014). Microstructural alterations of WM in ASDs such as reduced fractional anisotropy (FA), increased mean diffusivity (MD), and radial diffusivity (RD) in diffusion tensor imaging (DTI) representing abnormal myelination (Travers et al. 2015) or reduced coherence are speculated to be a cause of altered long-distance connectivity (Gibbard et al. 2013; Travers et al. 2015; Libero et al. 2016). Thus, structural models are essential in support of the analysis of FC using functional metrics, including fMRI. However, there has been no report evaluating FC and microstructural alterations in WM in ASD. To this end, we hypothesized that by combining diffusion MRI with fMRI, an anatomical perspective using diffusion MRI could be added to the network model obtained by fMRI. Here, we evaluated FC in patients with adult ASD, using resting-state functional MRI (rs-fMRI) and a new technique called diffusional kurtosis imaging (DKI).

DKI is one of the state-of-the-art sequences that can be used to describe and sensitively detect microstructural, developmental, and pathological changes in living tissue based on the non-Gaussian water-molecule theory and is more appropriate for complex brain tissues analyses than conventional DTI (Jensen et al. 2005; Hui et al. 2008; Fieremans et al. 2013; Umesh Rudrapatna et al. 2014; Zhu et al. 2015). DTI assumes that water diffusion has a Gaussian probability distribution, but barriers to diffusion, such as cell membranes and organelles, cause most diffusion processes in living tissues to follow a non-Gaussian distribution. Previous DTI reports regarding ASD have had conflicting results (Pugliese et al. 2009; Thomas et al. 2011; Pardini et al. 2012; Gibbard et al. 2013; Travers et al. 2015; Jou et al. 2016; Libero et al. 2016), suggesting some limitation of DTI (Alexander et al. 2007; Fieremans et al. 2011; Hattori et al. 2019). Reduced (Travers et al. 2015) or increased FA (Pardini et al. 2012); increased MD, RD, and AD (Travers et al. 2015); or no differences in the WM (Pugliese et al. 2009; Thomas et al. 2011; Jou et al. 2016) compared with HC have previously been reported. DTI indices suffer from reduced accuracy in regions with crossing fibers (Fieremans et al. 2011) and poor specificity with regard to pathology because pathology, including differences in axonal fiber density and caliber, myelination, and fiber tract homogeneity, might change the diffusional directionality according to the underlying structures (Alexander et al. 2007; Wheeler-Kingshott and Cercignani 2009). On the other hand, kurtosis values, albeit not specific with regard to the direction of the nerve fiber, reflect the microstructural complexity of the target tissue, and reduced mean kurtosis (MK), axial kurtosis (AK), and radial kurtosis (RK) reflect decreased microstructural complexity or heterogeneity within the brain tissue in all directions, in the axial direction of maximal diffusion, and in the perpendicular direction of maximal diffusion, respectively (Jensen et al. 2005; Jensen and Helpern 2010). Increased DKI measures suggest higher tissue complexity or greater hindrance to the diffusion of water (Jensen et al. 2005; Jensen and Helpern 2010; Steven et al. 2014).

## Methods

### Subjects

We recruited 33 patients with ASD who received outpatient treatment at Juntendo Koshigaya Hospital in Saitama, at Juntendo Shizuoka Hospital in Shizuoka, and at associated psychiatric clinics of Juntendo University in Tokyo, Japan. The patient group included 23 men and 10 women who ranged in age from 19 to 52 years old (mean age, 33.5 ± 8.8 years). We also recruited 33 age- and sex-matched healthy controls (HCs) at the same hospitals who had no history of neurological disorders. The HC group included 23 men and 10 women who ranged in age from 20 to 49 years old (mean age, 34.4 ± 8.3 years) (Table 1). All participants were right-handed, and all participants were paid for their involvement in the experiments. This study was approved by the ethics committees of Juntendo Koshigaya Hospital and Juntendo Shizuoka Hospital, and all participants provided written informed consent prior to inclusion in the study.

**Table 1:** Profile of sample groups.

	HC	ASD	Statistics
Number	33	33	
Age, mean ± SD (years)	34.4 ± 8.3	33.5 ± 8.8	F(1,64) = 0.09, *P* = 0.678
Gender (male/female)	23/10	23/10	
Handedness (left/right)	0/33	0/33	
Years of education, mean ± SD (years)	15.3 ± 2.4	14.6 ± 1.9	F(1,64) = 2.59, *P* = 0.189
AQ total score, mean ± SD AQ sub scores, mean ± SD Social skill Attention switch Attention Communication Imagination	15.5 ± 5.8 2.5 ± 2.1 3.5 ± 1.8 4.5 ± 2.3 1.9 ± 1.8 3.1 ± 1.7	32.7 ± 5.6 7.0 ± 1.6 7.4 ± 1.7 5.0 ± 1.9 7.2 ± 2.0 6.0 ± 2.0	F(1,64) = 0.15, *P* < 0.001 F(1,64) = 3.00, p < 0.001 F(1,64) = 0.10, p < 0.001 F(1,64) = 1.51, *P* = 0.332 F(1,64) = 0.22, p < 0.001 F(1,64) = 1.40, p < 0.001
SQ, mean ± SD	22.9 ± 11.2	26.7 ± 14.1	F(1,64) = 3.51, *P* = 0.23
EQ, mean ± SD	36.4 ± 10.0	24.3 ± 9.1	F(1,64) = 0.24, *P* < 0.001

HC, healthy control; ASD, autism spectrum disorder; AQ, autism-spectrum quotient; SQ, systemizing quotient; EQ, empathy, quotient; SD, standard deviation

The Diagnostic and Statistical Manual of Mental Disorders 5 (DSM-5) (American Psychiatric Association 2013) diagnosis was determined for each patient by use of a structured psychiatric interview and by reviewing the patients’ medical charts. None of the patients had undergone electroconvulsive shock treatment, and all were deemed to be in good physical health based on physical examination, laboratory testing, and medical history. The enrolled patients had no history of neurological illness affecting the central nervous system, addiction, or abuse of alcohol or other drugs as defined by the DSM-5 criteria (American Psychiatric Association 2013). All participants were confirmed to have no abnormal morphological finding by T1-weighted three-dimensional MRI in the experimental session.

To assess clinical symptoms, scores for Japanese versions (Kurita et al. 2003; Wakabayashi et al. 2006) of the autism-spectrum quotient (AQ) (Baron-Cohen et al. 2001), systemizing quotient (SQ) (Baron-Cohen et al. 2003), and empathizing quotient (EQ) (Baron-Cohen and Wheelwright 2004) were obtained for both groups. The AQ, EQ, and SQ are self-reported questionnaires consisting of 50 scored items, 60 items (40 scored items and 20 fillers), and 60 items (40 scored items and 20 fillers), respectively. The Empathizing-Systemizing theory (E-S theory) (Baron-Cohen 2009, 2010; Greenberg et al. 2018) suggests that cognitive styles (empathizing and systemizing) affect the development of various abilities such as social and physical causality. The E-S theory provides an understanding of the complex mechanisms involved with ASD (Kawata et al. 2014). Individuals with ASD show extremely high systemizing and low empathizing (Baron-Cohen et al. 2003; Baron-Cohen and Wheelwright 2004).

### rs-fMRI

#### Image acquisition and processing

Subjects were in a resting state with their eyes closed during data collection. Blood oxygen level–dependent (BOLD) fMRI data acquisition was performed at Juntendo University Hospital using a 3T MRI scanner (Achieva, Philips Healthcare, Best, the Netherlands) with a T2^*^-weighted gradient-echo echo-planar imaging (EPI) sequence. The parameters were as follows: echo time (TE) 30 ms, repetition time (TR) 2000 ms, field of view (FOV) 240 × 240 mm^2^, matrix 64 × 64, flip angle 90°, 22 axial slices, and voxel size 3.75 × 3.75 × 4.00 mm^3^. Each session consisted of a total of 200 scans, and the total image acquisition time was 6 min 40 s.

After discarding the first four volumes, 296 volumes were preprocessed using the CONN toolbox (www.nitrc.org/projects/conn, RRID:SCR_009550) (Whitfield-Gabrieli and Nieto-Castanon 2012) running on MATLAB version 8.3.0 (MathWorks, Inc., 2014). Slice timing was corrected based on slice order, and fMRI data were realigned and normalized in accordance with the standard Montreal Neurological Institute (MNI) template using the Statistical Parametric Mapping (SPM) Software platform (Wellcome Trust Centre for Neuroimaging, London, United Kingdom; http://www.fil.ion.ucl.ac.uk/spm/). The Artifact Detection Tools (ART) scrubbing procedure (www.nitrc.org/projects/artifact_detect/) was applied to minimize image artifacts due to head movement. The fMRI data were bandpass-filtered at 0.008 to 0.09 Hz, and the signal contributions from cerebrospinal fluid, white brain matter, and micro head movements (six motion parameters) were defined. Finally, a Gaussian filter kernel (full width at half maximum [FWHM] = 8 mm) was applied to spatially smooth all functional images.

#### Image analysis

This study analyzed the FC of the cortico-striatal network using seed-based FC analysis. As our specific interest was the caudate, we defined it as the seed. We used the CONN toolbox to analyze FC (Whitfield-Gabrieli and Nieto-Castanon 2012) and calculated Pearson’s correlation coefficients between the seed time-course and the time courses of all other voxels in the gray matter. This provided a seed-to-voxel connectivity matrix for individual analyses. Positive and negative correlation coefficients defined positive and negative FC, respectively (Whitfield-Gabrieli and Nieto-Castanon 2012). We then used Fisher’s transformation to convert the correlation coefficients to normally distributed scores that were used for population-level analysis. Between-group comparisons of the connectivity matrix were performed with the converted scores, followed by group-level estimates of the connectivity between regions of interest (ROIs) and evaluation of the between-group differences. Clusters were defined by the application of a high *t*-threshold of *P* < 0.001 (uncorrected) to individual voxels, while the threshold was *P* < 0.05 for the extracted clusters. The results were corrected by the false discovery rate (FDR).

### DKI

#### Image acquisition

Brain magnetic resonance imaging (MRI) of all participants was performed using the same 3T MRI scanner (Achieva, Philips) as rs-fMRI. Multishell diffusion-weighted (DW) MRI data were acquired with an echo-planar imaging (EPI) sequence consisting of two *b*-values (1,000 and 2,000 s/mm^2^) along 32 uniformly distributed directions in the anteroposterior phase-encoding direction. Each DW-MRI acquisition was rectified with a gradient-free image (*b* = 0 s/mm^2^). Standard and reverse phase–encoded blipped images with no diffusion weighting were also acquired to correct magnetic susceptibility-induced distortions that were affected by EPI acquisitions (Blip Up and Blip Down). Optimal conditions for the precise calculation of DKI parameters require at least three distinct *b*-values, including one *b* = 0 and 15 diffusion gradient directions. Further, Jensen et al. proposed the adoption of 30 directions because the oversampling of diffusion directions might render estimation in DKI metrics insensitive to motion artifacts and half the number of vertices in the archetype of diffusion directions could perform sufficiently (Jensen et al. 2005). Therefore, in this study, DWI was obtained with two *b*-values (1,000 and 2,000 s/mm2) accompanied by *b* = 0 acquired along 32 uniformly distributed directions. Sequence parameters were as follows: echo time 100 ms, repetition time 9,810 ms, diffusion gradient pulse duration (δ) 26.4 ms, diffusion gradient separation (Δ) 50.6 ms, matrix size 128 × 128, flip angle 90°, field of view 256 × 256 mm, slice thickness 2 mm with no gap, and acquisition time 13 min.

### Processing of DW-MRI data

All datasets were checked visually for severe artifacts in each direction of the 32 different axial, sagittal, and coronal directions. The EDDY and TOPUP toolboxes (Andersson and Sotiropoulos 2016) were applied to every DWI dataset to correct for susceptibility-induced geometric distortions, eddy current distortions, and intervolume subject motion (Andersson and Sotiropoulos 2016). Thereafter, diffusional kurtosis parameters were calculated using the diffusional kurtosis estimator (Tabesh et al. 2011) implemented in MATLAB (MathWorks, Natick, MA, USA) from which voxel-wise maps of MK, AK, and RK were then computed and generated. The ordinary least squares method was applied to the DW-MRI images with *b* = 0 and 1,000 s/mm^2^ to estimate the conventional diffusion tensor. After the estimation of the diffusion tensor, FA, MD, RD, and axial diffusivity (AD) were calculated based on the standard formulae (Basser et al. 1994).

Because of delays in adopting EDDY and TOPUP toolboxes for earlier image acquisition of our sampling, data of only 18 (13 males 5 females, age from 19 to 46 years old, mean age 29.1 ± 7.0 years) of 33 ASD patients and 17 (16 males 1 females, age from 23 to 49 years old, mean age, 32.7 ± 8.5 years) of 33 HCs (Table 2) were processed by these toolboxes and statistically assessed in the next steps. There were no differences in profiles between the whole cohort and the sample of individuals with data processed for DKI analysis, except for years of education, with individuals with ASD having fewer.

**Table 2:** Profile of sample groups of individuals with data processed for DKI analysis.

	HC	ASD	Statistics
Number	17	18	
Age, mean ± SD (years)	32.7 ± 8.5	29.1 ± 7.0	F(1,33) = 1.63, *P* = 0.179
Gender (male/female)	16/1	13/5	χ^2^ (1) = 0.01, *P* = 0.086
Handedness (left/right)	0/17	0/18	
Years of education, mean ± SD (years)	16.2 ± 2.1	14.4 ± 1.6	F(1,33) = 0.88, *P* = 0.005
AQ total score, mean ± SD AQ sub scores, mean ± SD Social skill Attention switch Attention Communication Imagination	15.8 ± 5.6 2.7 ± 2.2 3.3 ± 2.0 4.8 ± 2.6 1.8 ± 1.6 3.2 ± 1.7	32.8 ± 6.3 6.8 ± 1.8 7.9 ± 1.4 5.7 ± 1.5 6.7 ± 2.2 5.7 ± 2.2	F(1,33) = 0.46, *P* < 0.001 F(1,33) = 1.36, *P* < 0.001 F(1,33) = 1.75, *P* < 0.001 F(1,33) = 4.56, *P* = 0.254 F(1,33) = 1.07, *P* < 0.001 F(1,33) = 1.78, *P* = 0.001
SQ, mean ± SD	27.1 ± 12.7	30.7 ± 14.7	F(1,33) =0.69, *P* = 0.44
EQ, mean ± SD	37.8 ± 10.5	26.2 ± 10.2	F(1,33) = 0.01, *P* = 0.002

HC, healthy control; ASD, autism spectrum disorder; AQ, autism-spectrum quotient; SQ, systemizing quotient; EQ, empathy, quotient; SD, standard deviation.

#### Tract-based spatial statistics analysis

Whole brain voxel-wise statistical analyses of DTI/DKI parameters were performed using a voxel-wise tract-based spatial statistics (TBSS) package (Smith et al. 2006) implemented in FMRIB software library 5.0.9 (FSL, Oxford Centre for Functional MRI of the Brain, UK; www.fmrib.ox.ac.uk/fsl). For all participants, FA maps were nonlinearly warped to a template FA map, namely, FMRIB-58, in the Montreal Neurological Institute standard space by using the FMRIB nonlinear image registration tool, followed by visual inspection to confirm registration quality (Jenkinson et al. 2012). A mean FA map was then created by averaging the registered FA images, and the mean FA map was subsequently thinned to generate a mean FA skeleton of WM tracts. Next, the FA maps of each participant were projected onto the mean FA skeleton. This was done by filling the skeleton with FA values from the nearest tract center by searching perpendicularly to the local skeleton structure for the maximum FA value with a lower threshold of FA = 0.2, to exclude peripheral tracts and gray matter. The registration and projection parameters derived from the FA analysis were then applied to the other diffusion DTI/DKI parametric maps of each participant. The anatomical locations of the regions with significant group differences or correlations in DTI/DKI parameters on the WM skeleton were identified using JHU DTI-based WM atlases (Mori et al. 2005).

### ROI analysis

ROI analysis was performed on each cluster that was found to be significant in analyses for group differences or correlational TBSS analysis. The average diffusion metrics of each cluster were then measured.

### Statistical analysis

Statistical analyses were performed using IBM SPSS Statistical Package for the Social Sciences for Windows (release 21.0; IBM Corp., Armonk, NY, USA), except for general linear model analyses, which were performed using FSL. Demographic and clinical continuous variables were analyzed using the Mann–Whitney U test, whereas categorical variables were analyzed using the chi-square test. The threshold for statistical significance was set at *P* < 0.05.

Voxel-wise statistical analysis across participants on the skeleton was performed using the randomize tool, a component of FSL (Winkler et al. 2014), to ascribe a family-wise error-corrected *P* value (*P*_FWE_) to each cluster of voxels comprising the WM skeleton. The threshold-free cluster enhancement option was used in the randomize tool to avoid the selection of an arbitrary cluster-forming threshold, and 5000 permutations were performed to provide an empirical null distribution of maximal cluster size (Smith and Nichols 2009). Comparisons between the ASD and HC groups were performed using a general linear model framework with age and sex as covariates, and using the randomize tool with nonparametric permutation testing. The randomize tool was also used to examine the relationship between diffusion metrics and personality scores using multiple linear regression analysis. The significance threshold was determined with a *P*_FWE_ < 0.05. Because we found a discrepancy in terms of ROIs in WM that demonstrated significant differences in the comparisons and correlation analyses, we further analyzed the comparisons between the ASD and HC groups using an uncorrected *P* value of 0.05.

The unpaired Student’s *t*-test was used to assess between-group differences in average diffusion metrics of the clusters that showed significant group differences in the TBSS analysis. Furthermore, average diffusion metrics of the significant clusters were then correlated with personality scores using Spearman’s rank correlation test, with a significance threshold of *P* < 0.05. Considering the exploratory nature of this analysis, Bonferroni correction was not applied in Student’s *t*-test and Spearman’s rank correlation test.

## Results

### AQ, EQ, and SQ

**Fig. 1:**
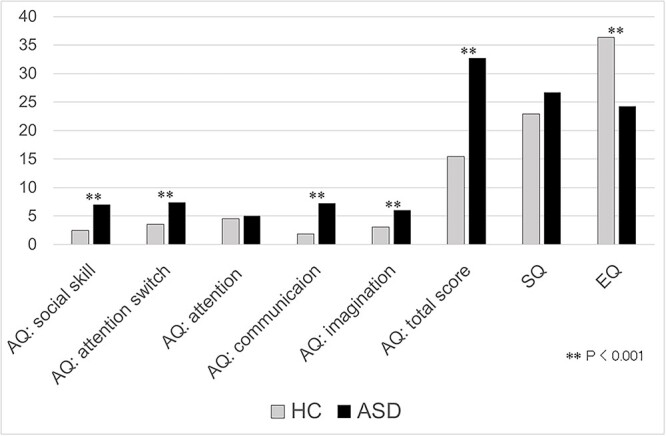
3Q profile of ASD and HC. Individuals with ASD showed higher AQ scores (*P* < 0.001) and lower EQ scores (*P* < 0.001) compared with HC. The patients had higher scores on AQ subcategories including “social skill” (*P* < 0.001), “attention switch” (*P* < 0.001), “communication” (*P* < 0.001), and “imagination” (*P* < 0.001). There were no significant differences between the groups in the SQ and AQ subcategory “attention.” AQ, autism-spectrum quotient; SQ, systemizing quotient; EQ empathy, quotient.

Individuals with ASD scored higher for AQ (*P* < 0.001) and lower for EQ (*P* < 0.001) compared with HC. The patients had higher scores on AQ subcategories including “social skill” (*P* < 0.001), “attention switch” (*P* < 0.001), “communication,” (*P* < 0.001), and “imagination” (*P* < 0.001). There were no significant differences between the groups in the SQ and AQ subscore “attention” (Table 1, Fig. 1).

### rsfMRI

**Fig. 2:**
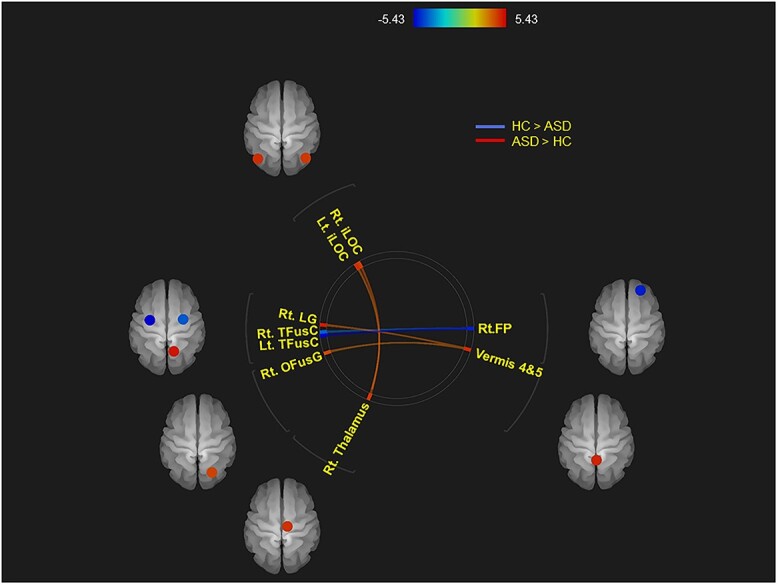
Clustered ROI-to-ROI functional connections with significant between-condition differences based on the criteria of *P*_FDR_ < 0.05. Individuals with ASD showed attenuated FC between the right FP and the bilateral TFusC, and enhanced FC between the right thalamus and the bilateral iLOC, and between the cerebellar vermis regions 4 and 5 and the right OFusG and the right LG, compared with HC (*P*_FDR_ < 0.05). As regards FC between the right thalamus and the bilateral iLOC, individuals with ASD, in turn, had attenuated negative FC. FP, frontal pole; TFusC, temporal fusiform cortex; iLOC, inferior division of lateral occipital cortex; OFusG, occipital fusiform gyrus; LG, lingual gyrus; *P*_FWE,_ family-wise error-corrected *P* value.

**Fig. 3:**
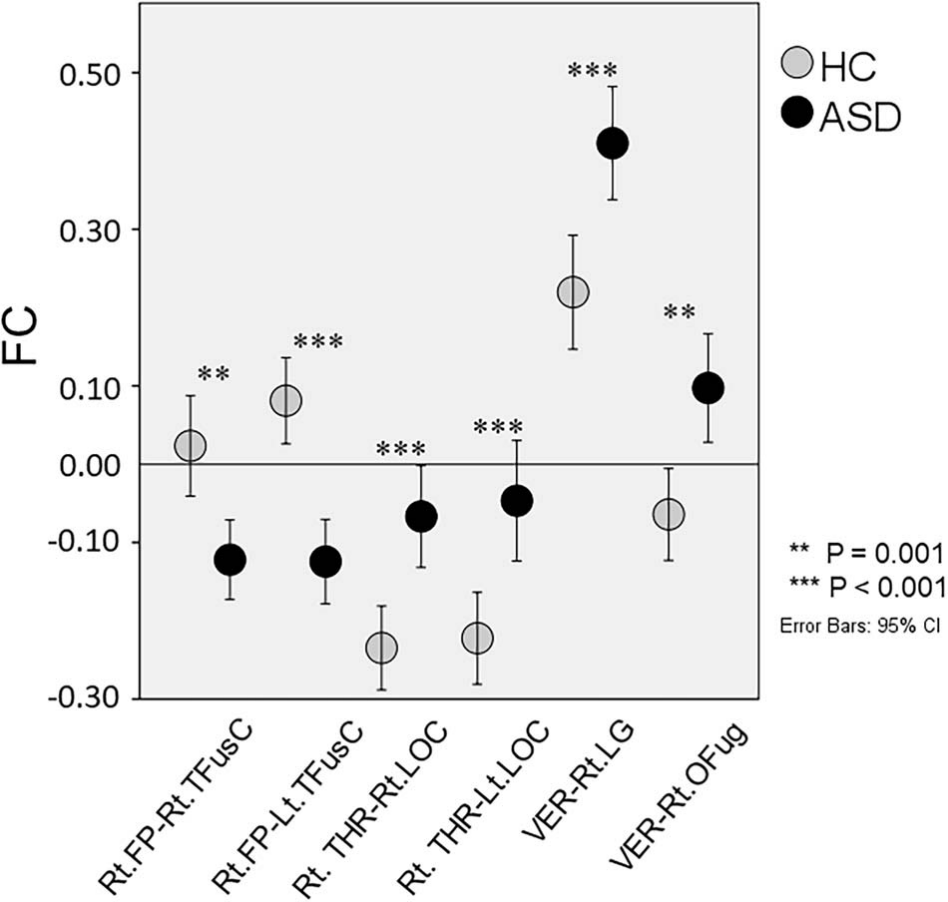
Quantitative measurements of FC among ROIs with significant differences. Individuals with ASD showed attenuated FC between the right FP and the bilateral TFusC, and enhanced FC between the right thalamus and the iLOC, and between the cerebellar vermis regions 4 and 5 and the right OFusG and the right LG, compared with HC. FP, frontal pole; TFusC, temporal fusiform cortex; iLOC, inferior division of lateral occipital cortex; OFusG, occipital fusiform gyrus; LG, lingual gyrus.

**Fig. 4:**
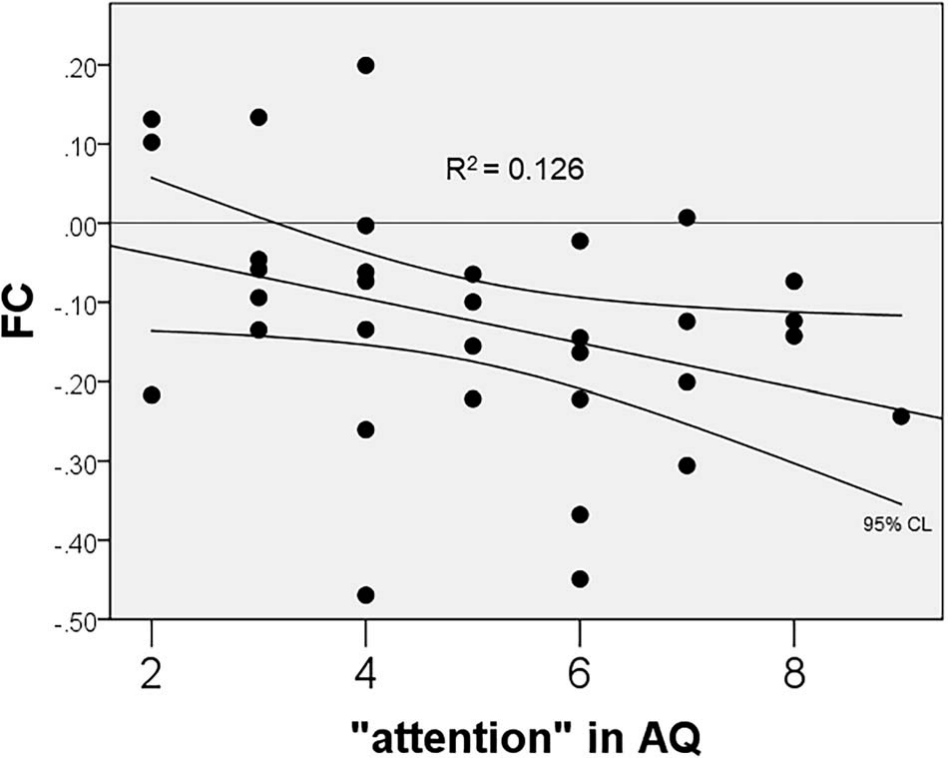
Correlation between FC (between the right frontal pole and the left temporal fusiform cortex) and the AQ subscore “attention” in ASD. In ASD, FC between the right frontal pole and the left temporal fusiform cortex corelated negatively with the AQ subscore “attention.”

Individuals with ASD showed attenuated FC between the right frontal pole (FP) and the bilateral temporal fusiform cortex (TFusC), and enhanced FC between the right thalamus and the bilateral inferior division of lateral occipital cortex (iLOC), and between the cerebellar vermis regions 4 and 5 (ROIs defined by CONN software; https://web.conn-toolbox.org/) (Nieto-Castanon 2020) and the right occipital fusiform gyrus (OFusG) and the right lingual gyrus (LG), compared with HC (*P* < 0.05, FDR) (Figs. 2 and 3). As regards FC between the right thalamus and the bilateral iLOC, ASD, in turn, had attenuated negative FC. In ASD, FC between the right FP and the left TFusC correlated negatively with the AQ subscore “attention” (Spearman *r* = −0.388, *P* < 0.05) (Fig. 4).

### DKI

**Fig. 5:**
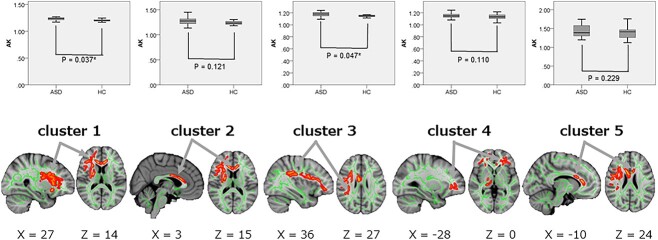
Comparison of AK between ASD and HC groups. TBSS (lower row) show significantly increased AK in five clusters (regions shown in Table 3) of WM in ASD compared with HC, and ROI (upper row) analyses supported the results of TBSS in cluster 1 and 3. In TBSS, thickned and embossed voxels represent higher AK (*P*_FWE_ < 0.05), and arrows show the peak of each cluster. The FA skeleton with an FA > 0.2 is shown in green. To facilitate visualization, the representations of the results are thickened using the fill script implemented in FSL. ^*^P < 0.05 in ROI analysis. AK, axial kurtosis; ASD, autism spectrum disorders; FA, fractional anisotropy; FSL, FMRIB software library; HC, healthy control; *P*_FWE,_ family-wise error-corrected *P* value; ROI, range of interest; TBSS, tract-based spatial statistic.

**Fig. 6:**
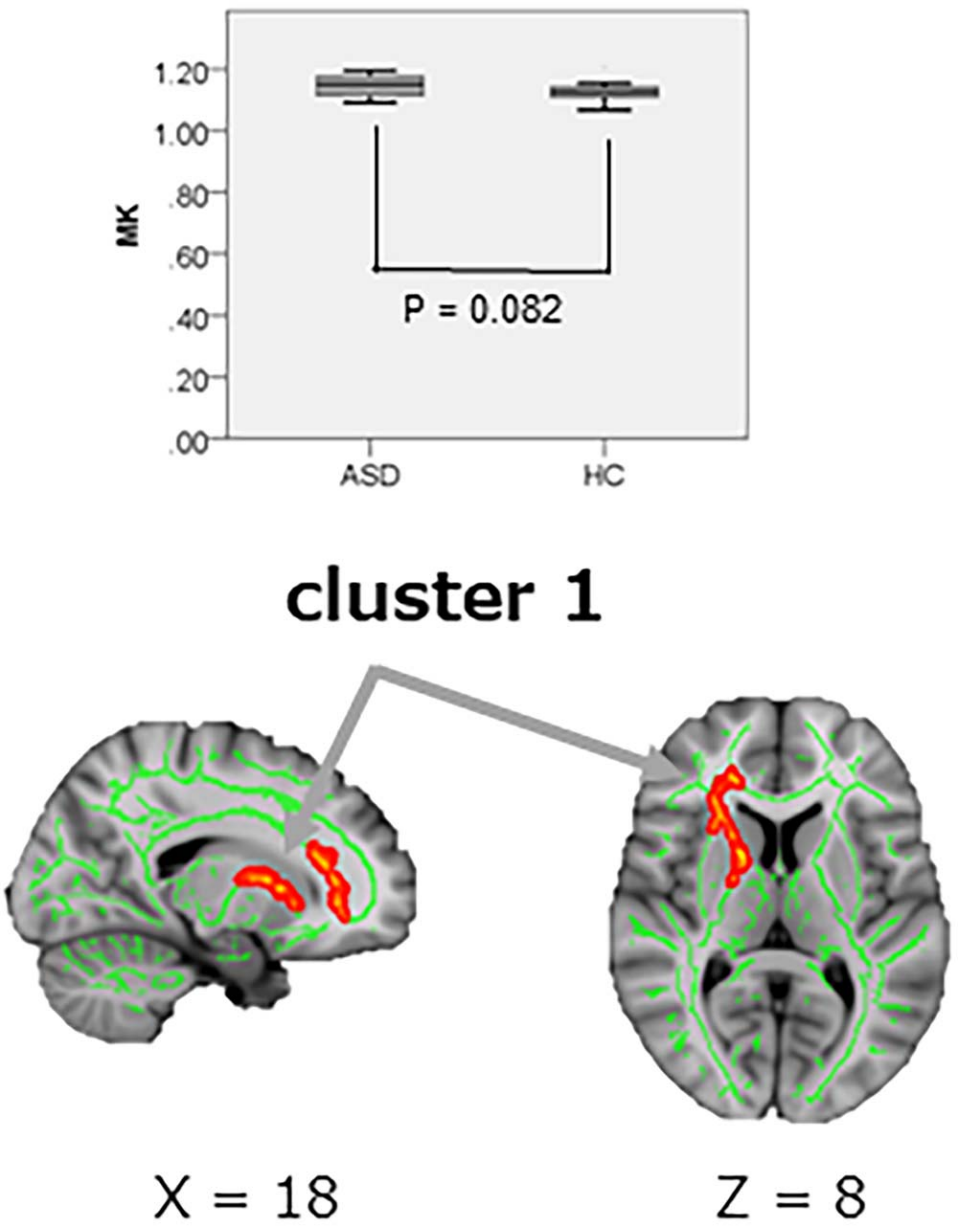
Group comparison of MK. TBSS (lower row) show significantly increased MK in one cluster (regions shown in Table 4) of WM in ASD compared with HC, although ROI (upper row) analysis did not reach significance. In TBSS, thickned and embossed voxels represent higher AK (*P*_FWE_ < 0.05), and arrows show the peak of each cluster. The FA skeleton with an FA > 0.2 is shown in green. To facilitate visualization, the representations of the results are thickened using the fill script implemented in FSL. MK, mean kurtosis; ASD, autism spectrum disorder; FA fractional anisotropy; FSL, FMRIB software library; HC, healthy control; *P*_FWE,_ family-wise error-corrected *P* value; ROI, range of interest; TBSS, tract-based spatial statistic.

TBSS analysis demonstrated significantly increased (*P*_FWE_ < 0.05) AK in the five clusters in ASD than in HC. The mean AK in clusters 1 and 3, including the right anterior corona radiata (ACR), forceps minor (FM), and right superior longitudinal fasciculus (SLF), supported the results of TBSS, where AK was significantly greater in the ASD than in the HC group (cluster 1, *P* = 0.037, Cohen’s *d* = 0.76; cluster 3, *P* = 0.047, Cohen’s *d* = 0.72), although the between-group differences in the other clusters (2, 4, and 5) were not significant (Fig. 5, Table 3). TBSS analysis also demonstrated significantly increased (*P*_FWE_ < 0.05) MK in one cluster, including the right ACR, right anterior limb of the internal capsule (IC), and right anterior thalamic radiation (ATR), in ASD than in HC, although the between-group differences in the mean MK in the cluster did not reach significance (Fig. 6, Table 4). No significant differences were observed in DTI (FA, MD, AD, and RD) or RK.

**Table 3:** Analysis of AK via tract-based spatial statistics and ROI analysis in the ASDs and HCs (Fig. 5).

Contrast	Cluster no.	WM areas	Number of voxels	MNI Coordinates ____________ X Y Z	Peak *T*-value	ASD group Mean AK ± SD	HC group Mean AK ± SD	*P*-value	Cohen’s *d*
ASD > HC	1 2 3 4 5	Rt. anterior corona radiata Forceps minor ^a^1 Genu of corpus callosum Forceps minor ^b^2 Rt. superior longitudinal fasciculus Lt. anterior corona radiata ^c^3 Body of corpus callosum	4279 925 611 549 52	99,144 93 123,170 67 38,100,105 80,125,101 72,120 79	4.27 6.62 3.67 4.09 2.54	1.23 ± 0.03 1.27 ± 0.08 1.18 ± 0.04 1.15 ± 0.04 1.43 ± 0.15	1.19 ± 0.07 1.21 ± 0.12 1.14 ± 0.05 1.12 ± 0.07 1.35 ± 0.20	0.037 0.121 0.047 0.110 0.229	0.76 0.55 0.72 0.57 0.43

AK, axial kurtosis; ASD, autism spectrum disorder; MNI, Montreal Neurological Institute; HC, healthy control; SD, standard deviation; WM, white matter.

^a^1, cluster 1 includes portions of the Rt. superior corona radiata, Rt. external capsule, Rt. anterior limb of internal capsule, Rt. posterior limb of internal capsule, Rt. cerebral peduncle, Rt. superior fronto-occipital fasciculus, Rt. inferior fronto-occipital fasciculus, Rt. superior longitudinal fasciculus, Rt. corticospinal tract, Rt. anterior thalamic radiation, and Rt. uncinate fasciculus, as well.

^b^2, cluster 2 includes portions of the body of corpus callosum and bilateral anterior corona radiata, as well.

^c^3, cluster 3 includes portions of the Lt. Inferior fronto-occipital fasciculus, forceps minor, and Lt. anterior thalamic radiation, as well.

**Table 4:** Analysis of MK via tract-based spatial statistics and ROI analysis in the ASDs and HCs (Fig. 6).

Contrast	Cluster no.	WM areas	Number of voxels	MNI coordinates ___________ X Y Z	Peak *T*-value	ASD group Mean MK ± SD	HC group Mean MK ± SD	*P*-value	Cohen’s *d*
ASD > HC	1	Rt. anterior corona radiata Rt. anterior limb of internal capsule Rt. anterior thalamic radiation ^a^1,	1,500	71,160 93	3.71	1.15 ± 0.03	1.13 ± 0.03	0.082	0.63

MK, mean kurtosis; ASD, autism spectrum disorder; MNI, Montreal Neurological Institute; HC, healthy control; SD, standard deviation; WM, white matter.

^a^1, cluster 1 includes portions of the forceps minor, genu of corpus callosum, Rt. superior corona radiata, Rt. posterior limb of internal capsule, Rt. superior fronto-occipital fasciculus, Rt. inferior front-occipital fasciculus, and Rt. external capsule, as well.

**Fig. 7:**
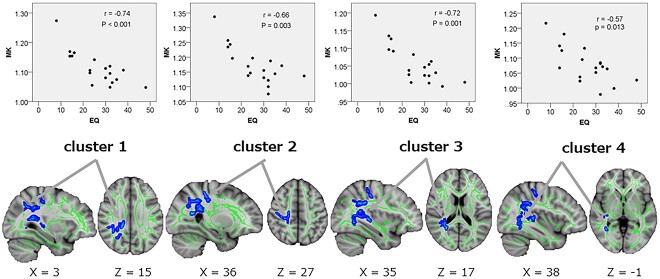
Correlation between MK and EQ in ASD. TBSS (lower row) and Spearman’s correlation test for mean MK (upper row) show significant negative correlations between MK and EQ in ASD in four clusters (regions shown in Table 5) of white matter. In TBSS, thickned and embossed voxels represent negative correlation (*P*_FWE_ < 0.05), and arrows show the peak of each cluster. The FA skeleton with an FA > 0.2 is shown in green. To facilitate visualization, the representations of the results are thickened using the fill script implemented in FSL. MK, mean kurtosis; EQ, empathy quotient; ASD, autism spectrum disorders; FA fractional anisotropy; FSL, FMRIB software library; TBSS, tract-based spatial statistic; *P*_FWE,_ family-wise error-corrected *P* value.

**Fig. 8:**
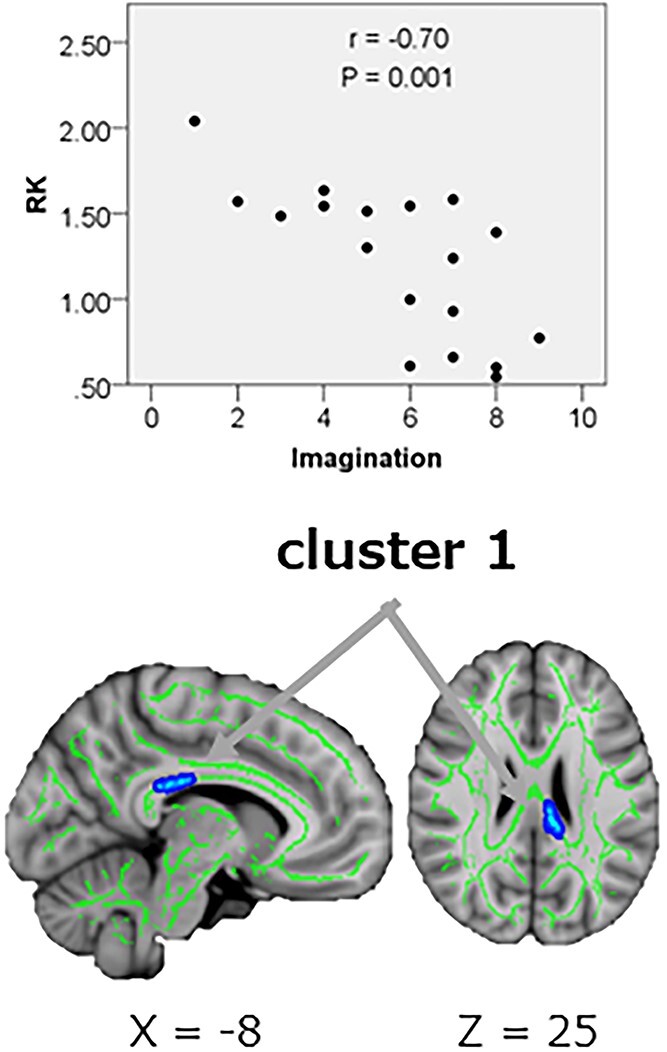
Correlation between RK and the AQ subscore “imagination” in ASD. TBSS (lower row) and Spearman’s correlation test for mean RK (upper row) show significant negative correlations between RK and the AQ subscore “imagination” in ASD in one cluster (regions shown in Table 5) of white matter. In TBSS, thickend and embossed voxels represent negative correlation (*P*_FWE_ < 0.05), and arrows show the peak of each cluster. The FA skeleton with an FA > 0.2 is shown in green. To facilitate visualization, the representations of the results are thickened using the fill script implemented in FSL. RK, radial kurtosis; AQ, autism-spectrum quotient; ASD, autism spectrum disorder; FA fractional anisotropy; FSL, FMRIB software library; TBSS, tract-based spatial statistic; *P*_FWE,_ family-wise error-corrected *P* value.

TBSS analysis showed a significant negative correlation (*P*_FWE_ < 0.05) between MK and EQ of four clusters in the WM in ASD. The four clusters were as follows: cluster 1: the right SLF and the splenium of the corpus callosum (CC); cluster 2: the right corticospinal tract and the right superior corona radiata; cluster 3: the right posterior corona radiata and right retrolenticular part of the IC; and cluster 4: the right posterior thalamic radiation. Furthermore, RK correlated negatively with AQ subscore “imagination” in one cluster of the WM in ASD. The cluster included the body of the CC and the splenium of the CC. Spearman’s rank correlation test for the mean MK and RK in each cluster also confirmed the findings of the TBSS analyses (Table 5, Figs. 7 and 8).

**Table 5:** Correlations between MK & RK and personality scales based on tract-based spatial statistics and ROI analyses (Figs 7 and 8).

Contrast of correlation	Cuister no.	WM areas	Number of voxels	MNI coordinates ____________ X Y Z	Peak *T*-value	MK Mean MK ± SD	Coefficient of correlation (*r*)	*P*-value
MK vs. EQ in ASDs	1 2 3 4	Rt. superior longitudinal fasciculus, Splenium of corpus callosum ^a^1 Rt. corticospinal tract, Rt. superior corona radiata ^b^2 Rt. posterior corona radiata, Rt. retrolenticular part of internal capsule ^c^3 Rt. posterior thalamic radiation ^d^4	1021 550 521 103	60 85,103 51,100,124 62 88 94 54 72 70	4.98 7.51 4.58 3.86	1.11 ± 0.06 1.17 ± 0.06 1.06 ± 0.05 1.08 ± 0.06	−0.74 −0.66 −0.72 −0.57	0.001> 0.003 0.001 0.013
RK vs. “imagination” in AQ in ASDs	1	Body of corpus callosum, splenium of corpus callosum	108	98,104 98	4.36	1.21 ± 0.44	−0.70	0.001

MK, mean kurtosis; RK, radial kurtosis; ASD, autism spectrum disorder; MNI, Montreal Neurological Institute; HC, healthy control; SD, standard deviation; WM, white matter.

^a^1, cluster 1 includes portions of the Rt. posterior corona radiata and body of corpus callosum.

^b^2, cluster 2 includes portions of the Rt. superior longitudinal fasciculus and body of corpus callosum.

^c^3, cluster 3 includes portions of the Rt. posterior thalamic radiation, Rt. superior longitudinal fasciculus, Rt. Inferior fronto-occipital fasciculus, splenium of corpus callosum, Rt. tapetum, and forceps major, as well.

^d^4, cluster 4 includes portions of the Rt. inferior fronto-occipital fasciculus, Rt. inferior longitudinal fasciculus, and Rt. sagittal stratum, as well.

### Correlation between rs-fMRI and DKI

**Fig. 9:**
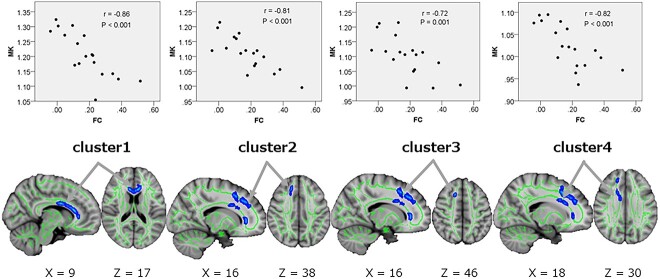
Correlation between MK and FC in rs-fMRI between the cerebellar vermis and the right occipital fusiform gyrus in ASD. TBSS (lower row) and Spearman’s correlation test for mean MK (upper row) show significant negative correlations between MK and FC in rs-fMRI between the cerebellar vermis and the right occipital fusiform gyrus in ASD in four clusters (regions shown in Table 6) of WM. In TBSS, blue to light-blue voxels represent negative correlation (*P*_FWE_ < 0.05), and arrows show the peak of each cluster. The FA skeleton with an FA > 0.2 is shown in green. To facilitate visualization, the representations of the results are thickened using the fill script implemented in FSL. MK, mean kurtosis; rs-fMRI, resting-state functional MRI; ASD, autism spectrum disorder; FA, fractional anisotropy; FSL, FMRIB software library; TBSS, tract-based spatial statistic; *P*_FWE,_ family-wise error-corrected *P* value.

TBSS analysis, additionally, showed a significant negative correlation between MK and FC between the cerebellar vermis regions 4 and 5 and the right OFusG of four clusters in the WM in ASD. The four clusters were as follows: cluster 1: the genu and body of the CC, FM; cluster 2: the FM; cluster 3: the right SLF; and cluster 4: the right ACR (Table 6, Fig. 9).

**Table 6:** Correlations between MK and FC in rs-fMRI between the cerebellar vermis and the right occipital fungiform gyrus in ASDs based on tract-based spatial statistics and ROI analyses (Fig. 9).

Contrast of correlation	Cluster no.	WM areas	Number of voxels	MNI coordinates __________ X Y Z	Peak *T*-value	MK Mean MK ± SD	Coefficient of correlation (*r*)	*P*-value
MK vs. FC on rs-fMRI in ASDs	1 2 3 4	Genu of corpus callosum, Forceps minor Body of corpus callosum Rt. anterior corona radiata Lt. cingulum cingulate gyrus Forceps minor Rt. inferior front- occipital fasciculus Rt. anterior thalamic radiation Rt. superior longitudinal fasciculus Rt. superior longitudinal fasciculus Temporal part of Rt. superior longitudinal fasciculus Rt. anterior corona radiata Body of corpus callosum Rt. superior corona radiata	892 171 120 89	101,151 90 74,162,107 72,143,125 68,153 94	4.78 6.30 3.62 5.22	1.11 ± 0.06 1.17 ± 0.06 1.06 ± 0.05 1.08 ± 0.06	−0.86 −0.81 −0.72 −0.82	0.001> 0.001> 0.001 0.001>

MK; mean kurtosis; rs-fMRI, resting-state functional MRI; FC, functional connectivity; ASD, autism spectrum disorder; MNI, Montreal Neurological Institute; HC, healthy control; SD, standard deviation; WM, white matter.

## Discussion

In this study, individuals with ASD showed attenuated FC between the right FP and the bilateral TFusC, and enhanced FC between the right thalamus and the bilateral iLOC, and between the cerebellar vermis and the right OFusG and the right LG, compared with HC. In ASD, FC between the right FP and the left TFusC correlated negatively with the AQ subscore “attention.” Individuals with ASD demonstrated significantly increased AK in the right ACR, FM, and right SLF than did HC. Individuals with ASD also demonstrated significantly increased MK in a cluster including the right ACR, right anterior limb of the IC, and right ATR in comparison with HC, although the between-group differences in the cluster did not reach significance. Individuals with ASD showed a significant negative correlation between MK and EQ in the right SLF, the splenium of the CC, the right corticospinal tract, right. Superior corona radiata, the right posterior corona radiata, right retrolenticular part of the IC, and the right posterior thalamic radiation. Furthermore, RK in ASD correlated negatively with the AQ subscore “imagination” in the body of the CC and the splenium of the CC. Individuals with ASD showed a significant negative correlation between MK and FC between the cerebellar vermis and the right OFusG in the genu and body of the CC, the FM, the right SLF, and the right ACR.

As regards our rs-fMRI findings, an enhanced cerebellar network and attenuated corticocortical connections in fronto-temporal cortices are in agreement with the previous reports (Khan et al. 2015; Nakamura et al. 2023). Furthermore, consistent with our results, thalamic-temporal and thalamic-occipital overconnectivity in ASD patients (Nair et al. 2013; Nair et al. 2015; Baran et al. 2023) or infants at familial high risk for ASD (Nair et al. 2021; Wagner et al. 2023) have been reported. Thalamocortical hyperconnectivity in ASD may indicate that decreased thalamic inhibition leads to an increase in less-filtered sensory information reaching the cortex where it disrupts attention and contributes to sensory sensitivity (Baran et al. 2023). To our knowledge, there has been no prior report of our finding that FC between the fusiform gyrus and the frontal pole is attenuated in ASD. Amygdala-fusiform/cerebellar underconnectivity is related to social and nonsocial cognition in adults with ASD (Goodwill et al. 2023). However, in contrast, overconnectivity encompassing the right Heschl’s and inferior temporal gyrus with a greater right-dominance asymmetric value correlates with symptom severity in preschoolers with ASD (Kim et al. 2021). In our data, FC correlated negatively with the AQ subscore “attention” in ASD, but HC and ASD had opposite polarities of FC related to the fusiform gyrus, suggesting that the strategy of information processing in ASD differs from that in HC, although clinical interpretation of the atypicality of FC between the fusiform gyrus and the frontal pole calls for further investigations.

Microstructural abnormalities of some WM tracts including the ATR, CC, cingulum, FC, forceps major, inferior fronto-occipital fasciculus, inferior longitudinal fasciculus, and SLF are reported to be associated with clinical phenotype autism (Ameis and Catani 2015; Andica et al. 2021). Of ROIs with significant alteration in this study, ATR is anatomically defined as a projection fiber that connects the dorsomedial thalamic nucleus to the prefrontal cortex through the anterior limb of the IC (Lazar et al. 2014; Koolschijn et al. 2017). The CC, defined as a commissural fiber, connects the cortices of the two cerebral hemispheres and is related to cognitive and social functions in ASD (Bakhtiari et al. 2012; Gibbard et al. 2013; Lazar et al. 2014; Catani et al. 2016; Nickel et al. 2017; Dimond et al. 2019; Hattori et al. 2019; Ohta et al. 2020). SLF, defined as a long-range associative fiber, connects the frontal lobe to the parietal lobe and partially to the temporal lobe and functions in visuospatial attention, language auditory comprehension, articulatory processing, reading, and lexical access (Bakhtiari et al. 2012; Gibbard et al. 2013; Lazar et al. 2014; Koolschijn et al. 2017; Dimond et al. 2019; Mohajer et al. 2019).

In the previous DKI studies, we reported lower AK in the body and splenium of the CC in ASD (Hattori et al. 2019). A decrease in AK, representing a decrease in complexity along the fiber direction, has been suggested to be associated with axonal degeneration (Jensen et al. 2005). Furthermore, we employed new tensor imaging methods NODDI (neurite density index [NDI], orientation dispersion index, and isotropic volume fraction [ISOVF]), and observed significantly lower NDI and higher ISOVF, suggestive of decreased neurite density and increased extracellular free water, reflecting neural loss and neuroinflammation respectively, mainly in commissural and long-range association tracts in ASD (Andica et al. 2021). Consistent with such NDI reduction, postmortem studies on the brains of individuals with ASD have previously reported reduced numbers of medium and large-caliber axons, which likely affect synaptic function (Zikopoulos and Barbas 2010; Wegiel et al. 2018). As assessed by DKI and fixel-based analysis (FBA) (Raffelt et al. 2017), reduced axonal density, marked by a decreased axonal water fraction, has also been suggested within the CC and long-range association tracts in adult ASD (Lazar et al. 2014; Dimond et al. 2019). Loss of axon integrity may result in reduced information-processing speed in ASD (Wegiel et al. 2018).

Inconsistent with our results, the previous DKI studies demonstrated decreased MK (McKenna et al. 2020; Tang et al. 2022), AK (Hattori et al. 2019; Tang et al. 2022), RK (McKenna et al. 2020; Tang et al. 2022), or no differences (He et al. 2022) in ASD compared with HC. Lower DKI metrics have been interpreted as developmental abnormalities, axon damage (He et al. 2022), differences in the microstructural environment such as decreased neuronal density (Irie et al. 2018) and size, atypically sized cortical columns, limited dendritic arborizations, or reduced complexity (McKenna et al. 2020).

These inconsistencies might be due to variations in acquisition parameters, sample size, (Hattori et al. 2019), or the ages of cohorts (Andica et al. 2021). Added to these, abnormalities in brain development in patients with ASD have been reported to be more extensive at younger ages than at older ages (Aylward et al. 2002). Studies investigating age-based trajectories have found overgrowth in early development followed by either degeneration or retention of abnormal connections in adolescence and adulthood. Studies of WM integrity and atypical connective patterns in different age groups have detected aberrations both in the cerebral cortex and in the cerebellum of adolescents with ASD, suggesting that arrested development may occur in late childhood or early adolescence (D'Mello and Stoodley 2015; Jouravlev et al. 2020; Olulade et al. 2020). For example, higher FA values in early childhood decreased with age, crossed the curve of HC, and plateaued below the FA values of the typically developing group in follow-up analyses (Travers et al. 2015). Reduced FA for the left superior longitudinal fasciculus in children with ASD increased with age (Libero et al. 2016). Moreover, a widespread cortical thickness increase was demonstrated in children with ASD compared with HC, whereas adults with ASD showed an increased rate of cortical thinning (Khundrakpam et al. 2017; Bethlehem et al. 2020). Speculatively, factors such as atypical early development or maintenance of myelin (occurring before birth or in the first few years of life) could underlie these brain differences in ASD (McKenna et al. 2020). The variability noted across studies is likely also due to the genetic and observed phenotypic diversity among diagnosed individuals and the differing ages of study participants (Sydnor and Aldinger 2022). Our sample is composed of a relatively older (33.5 ± 8.8 years) and wider (19 to 52 years) range of ages than previous DKI reports, including on pediatric (He et al. 2022; Tang et al. 2022) or adolescent ASD (McKenna et al. 2020). To our knowledge, however, there have been no reports directly assessing age-related differences of DKI measures in ASD. (As reference materials, the Spearman’s rank correlation coefficients between AK/MK and age in clusters where between-group differences were significant (Figs. 5 and 6, Tables 3 and 4) are as follows: HC, AK cluster 1: −0.464, cluster 2: −0.569^*^, cluster 3: 0.142, cluster 4: −0.441, cluster 5: −0.051, MK cluster 1: -0.137; ASD, AK cluster 1: 0.278, cluster 2: 0.368, cluster 3: 0.366, cluster 4: 0.503^*^, cluster 5: 0.340, MK cluster 1: 0.340 [^*^: *P* < 0.05]. Although these results are statistically weak, DKI measures may tend to increase with age in ASD, whereas they may decrease in HC.

MK is, in analogy with our results in some ROIs of WM, elevated in some pathological conditions, such as acute brain infarction (Hui et al. 2012) and high-grade glioma (Raab et al. 2010). Postmortem studies have demonstrated the presence of brain neuroinflammation in ASD, as shown by marked activation of astrocytes and microglia together with abnormal chemokine and cytokine levels (Vargas et al. 2005; Li et al. 2009; Matta et al. 2019; Liao et al. 2020).

Other than ASD, although some inconsistencies across reports remain, the use of DKI metrics as a sensitive diagnostic biomarker of Parkinson’s disease (PD) has been discussed (Wang et al. 2011; Kamagata et al. 2013; Huang et al. 2022; Li et al. 2022; Meng et al. 2023). Higher MK within the substantia nigra and basal ganglia in PD may be related to increased complexity due to loss of cells that leads to secondary gliosis (Wang et al. 2011) or to the loss of dopaminergic neurons, oxidative stress, and chronic inflammation that result in limited water molecule diffusion. From the perspective of compensation, early inflammation leads to an increase in the number of glial cells and cytokine activation; the increase of glia exceeds the loss of dopaminergic neurons. Thus, early neuroinflammation can effectively alleviate the degeneration of dopaminergic neurons (Deleidi and Gasser 2013).

Increased DKI metrics might represent neuroinflammation, increased complexity, or disrupted WM tissue integrity that alters long-distance connectivity. Such pathogenetic factors are in agreement with our result demonstrating a negative correlation between MK and EQ. On the other hand, protective or compensating adaptations of inflammation, as occur with PD, might lead to more abundant glial cells and cytokine activation effectively alleviating the degeneration of neurons, resulting in increased complexity. In line with our results, elevated kurtosis values in ASD, closer to the HC range, appear to be associated with fewer restrictive and repetitive behaviors and better social interaction skills (McKenna et al. 2020). Increased kurtosis in gray matter (GM) and its associated cellular processes may have a protective function or supporting effect, because increased MK in GM has been correlated with better performance on executive function tests in traumatic brain injury, multiple sclerosis, and schizophrenia (Grossman et al. 2013; Bester et al. 2015; Matta et al. 2019). These findings support our observation that RK negatively correlated with the AQ subscore “imagination.” Participants in our ASD cohort with a relatively older and wider range of ages have variable and heterogeneous microstructural alterations, represented by DKI metrics with complex polarity that reflects diverse atypical developing stages. In the light of the variability noted across studies and our results, directions of DKI measures in ASD might be diverse and heterogeneous across patients or be affected by age. This calls for further investigation to obtain an overarching hypothesis.

To our knowledge, no study has tried to investigate associations between microstructural alterations and aberrant FC in ASD. Our findings of significant correlations of DKI measures in commissural and long-range association tracts with cerebral cortical connectivity allow the interpretation of previous findings on changes in diffusion tensor metrics in the WM and pathological FC of ASD. In the CC, FM, SLF, and ACR, MK significantly correlated with FC between the cerebellar vermis and the right OFusG, suggesting that the FC abnormality in ASD observed in rs-fMRI may be attributed to microstructural alterations of the commissural and long-range association tracts in the WM tracts indicated by DKI.

CC alteration in DTI (Alexander et al. 2007) and smaller volume (Frazier and Hardan 2009; Prigge et al. 2013) have been repeatedly shown in ASD, and functional imaging studies have demonstrated correlations between FC measures and the sizes of relevant regions of the CC (Just et al. 2007; Mason et al. 2008; Damarla et al. 2010; Schipul et al. 2012). Relevant parts of the CC, through which many of the bilaterally activated cortical areas communicate, were smaller in cross-sectional area in ASD, possibly implicating them in impaired information integration resulting from reduced intra-cortical connectivity (Just et al. 2007). Specifically, the CC is the largest WM tract in the brain and is known to reciprocally affect cortical development (Pietrasanta et al. 2012). This means that the CC could be intimately involved in the development of other structural brain differences observed in ASD (Travers et al. 2015). Atypical development of cortical thickness has been longitudinally observed in ASD (Zielinski et al. 2014).

Moreover, structural and functional abnormalities of the cerebellum and its altered connectivity with forebrain structures play roles beyond hallmark features of ASD (Brandenburg et al. 2022; Sydnor and Aldinger 2022; Wei et al. 2022; Zhu et al. 2022; Kumar et al. 2023; Lu et al. 2023; Nakamura et al. 2023). Along with our results, increased FC in the left posterior inferior temporal gyrus and anterior cerebellar lobes has been reported (Nakamura et al. 2023). Through its complex network of connections with cortical and subcortical brain regions, the cerebellum acts as a sensorimotor regulator and affects changes in executive and limbic processing, including motor and nonmotor learning, higher executive functions, affect regulation, language comprehension and production, social skills, visual–spatial performance, and working memory functions (Stoodley 2012; Starowicz-Filip et al. 2017; Hoche et al. 2018; Mapelli et al. 2022). FC studies have also found atypical lateralization patterns, including reduced lateralization of cerebellar connectivity, noncanonical activity in cerebrocerebellar networks related to social interaction and language, or increases in neurotypically out-of-network cerebellocortical functional activity. Individuals with ASD show a pattern of robustly increased connectivity compared with the HC for sensorimotor ROIs, but predominantly reduced connectivity for supramodal ROIs accompanied by significantly increased noncanonical connections (between sensorimotor cerebral and supramodal cerebellar ROIs and vice versa), supporting cerebellar participation in supramodal cognition (Khan et al. 2015). Many other aberrant connections have also been identified in ASD, including additional connections of classically considered nonmotor areas of the cerebellum to sensorimotor cerebral cortices: particularly, regions of the occipital lobe, premotor and primary motor cortices, and primary somatosensory cortex (Khan et al. 2015). Atypical eye gaze (Ma et al. 2021), delayed orienting (Townsend et al. 1996), impairments in smooth pursuit (Takarae et al. 2004), altered movement perception, and deficits in facial perception (Griffin et al. 2021) demonstrated by individuals with ASD (Johnson et al. 2016; Chung and Son 2020) are likely mediated by these abnormal sensorimotor connections, other alterations in olivofloccular circuitry (Wegiel et al. 2013), and altered Purkinje cells activity and number (Sydnor and Aldinger 2022). The connective abnormalities often present as reduced lateralization of typically asymmetrical processes and could be the result of abnormal retention of early developmental connections or recruitment of extra computational power as compensation for developmental damage (Sydnor and Aldinger 2022).

ASD in our cohorts showed a significant negative correlation of FC between the right FP and the left TFusC with “attention” of AQ and negative correlations between MK and EQ in the right SLF and the splenium of the CC and between RK and “imagination” of AQ in the body of the CC and its splenium. In line with our results, decreased kurtosis in gray matter ROIs in adolescents with ASD correlated with increased repetitive and restricted behaviors and poor social interaction symptoms (McKenna et al. 2020). In children and adolescents with ASD, cerebellar connectivity to left cerebral sensorimotor and supramodal ROIs correlated positively or negatively with the Social Responsiveness Scale total scores and cerebellar connectivity with the right sensorimotor ROI was negatively correlated with nonverbal IQ (Khan et al. 2015). An increase in noncanonical cerebellocerebral and cerebrocerebellar connections is significantly correlated with increased ASD behaviors, although these changes in network activity often do not persist into adulthood (Sydnor and Aldinger 2022). Associations between rs-fMRI, DKI measures, and clinical assessments observed in our results support that ectopia from a canonical distribution of HC should represent the structural and functional contributions of WM to the pathophysiology of individuals with ASD, although their phenotypes are extremely heterogeneous and diverse in polarity.

Our results showed increased FC between the cerebellar vermis and the right OFusG and right LG. Further, FC between the cerebellar vermis and the right OFusG showed significant negative correlation with MK in the CC. Speculatively, increased FC between the cerebellum and occipital lobe might be a compensative extra connectivity. However, the clusters showing a significant negative correlation between FC and MK in the CC did not exactly match with that showing a group difference in MK. Thus, the pathology of the CC might include variable microstructural alterations, with noncanonical MK deviation from that common in neurotypical individuals. Any explanation for this negative correlation will be difficult to disentangle and have to be modest. One speculation is that patients with higher MK, which might represent a compensatively protective function or preserved axonal density, did not need to recruit noncanonical connectivity such as cerebellocortical connectivity. Another plausible interpretation of this negative correlation is that individuals with ASD with higher MK of the CC representing reduced WM integrity and increased complexity might have difficulties even in recruiting compensative extra connectivity. Further exploration is necessary to address this issue. Our findings never illuminate the complete picture of ASD except to indicate that the Gaussian distribution of water diffusion in WM is partially altered, accompanied by incomplete association with FC. Thus, the next frontier requires coordinating studies and a multimodal approach including histopathological confirmation and investigation of the genetic architecture of ASD to obtain a consensus on a uniform explanatory model of functional and structural brain atypicalities in ASD and elucidate the intrinsic pathogenetic pathway of the disease.

## Limitations

There are some potential limitations of our study. First, it had a small sample size, and not all data of the participants were processed for DKI analysis, because of a technical flaw in earlier image acquisition. More large multicenter datasets need to be assessed in the future to confirm the results. Second, we did not assess the intelligence of individuals with ASD, nor any pharmacological contamination administered to them. We also used only the self-reported questionnaires AQ, SQ, and EQ, not objective rating scales for symptom severity or social skills. Further studies should examine more extensive assessments of clinical symptoms and social status. Third, our results included some inconsistencies with those of previous studies, and diversity of directionality, which might have resulted from the wide age range of our ASD cohort. A nonlongitudinal study design such as ours is less sensitive to age-related changes during wide windows of development. Further progress will require a longitudinal cohort study to disentangle the complexity and heterogeneity of the pathology of this disease.

## Conclusion

We evaluated FC in adult ASD using rs-fMRI and DKI. In the CC, FM, SLF, and ACR, ASD showed significant correlations between rs-fMRI, DKI measures, and clinical assessments, suggesting that ectopia from a canonical distribution of HC should represent the structural and functional contribution of WM to the pathophysiology of ASD, although its phenotype exhibits both extreme heterogeneity and diversity in polarity. MK in ASD was elevated and significantly correlated with FC between the cerebellar vermis and the right OFusG, suggesting that the FC abnormality in ASD observed in rs-fMRI may be attributed to microstructural alterations of the commissural and long-range association tracts in the WM tracts indicated by DKI. We speculate that increased DKI metrics might represent neuroinflammation, increased complexity, or disrupted WM tissue integrity that alters long-distance connectivity. Nonetheless, protective or compensating adaptations of inflammation might lead to more abundant glial cells and cytokine activation effectively alleviating the degeneration of neurons, resulting in increased complexity.

## Author contributions

Yasuhito Nagai (Investigation, Project administration), Eiji Kirino (Conceptualization, Data curation, Formal analysis, Funding acquisition, Investigation, Methodology, Project administration, Writing—original draft, Writing—review & editing), Shoji Tanaka (Data curation, Investigation, Methodology), Chie Usui (Investigation, Resources), Rie Inami (Investigation), Reiichi Inoue (Project administration, Supervision) Aki Hattori), Investigation), Wataru Uchida (Data curation, Visualization), Koji Kamagata (Data curation, Methodology, Supervision), and Shigeki Aoki (Supervision)

## CRediT for author contributions

YN, EK, ST, RIno and SA conceived the present study and its methods. EK, ST, WU and KK conducted statistical analyses. YN and EK wrote the manuscript. YN, EK, CU and RIna recruited participants and were involved in clinical and diagnostic assessments. ST, AH, WU and KK analyze d MRI data and preparing the figures. KK and SA provided technical support for MRI and data processing. All authors contributed to and approved the final manuscript.

## Funding

This research was supported by grants from the Research Support Foundation 2017-2023 of the Juntendo Institute of Mental Health and by a Grant-in-Aid for Scientific Research KAKENHI (grant numbers 26461757 and 19K08026).

*Conflict of interest statement*: The authors declare no conflicts of interest.
